# Peripheral Blood T Cells of Patients with IPAH Have a Reduced Cytokine-Producing Capacity

**DOI:** 10.3390/ijms23126508

**Published:** 2022-06-10

**Authors:** Denise van Uden, Thomas Koudstaal, Jennifer A. C. van Hulst, Madelief Vink, Menno van Nimwegen, Leon M. van den Toorn, Prewesh P. Chandoesing, Annemien E. van den Bosch, Mirjam Kool, Rudi W. Hendriks, Karin A. Boomars

**Affiliations:** 1 Department of Pulmonary Medicine, Erasmus MC, University Medical Center Rotterdam, 3015 GD Rotterdam, The Netherlands; d.vanuden@erasmusmc.nl (D.v.U.); t.koudstaal.1@erasmusmc.nl (T.K.); j.vanhulst@erasmusmc.nl (J.A.C.v.H.); m.vink.1@erasmusmc.nl (M.V.); m.vannimwegen@erasmusmc.nl (M.v.N.); l.vandentoorn@erasmusmc.nl (L.M.v.d.T.); p.chandoesing@erasmusmc.nl (P.P.C.); m.kool@erasmusmc.nl (M.K.); 2 Department of Cardiology, Erasmus MC, University Medical Center Rotterdam, 3015 GD Rotterdam, The Netherlands; a.e.vandenbosch@erasmusmc.nl

**Keywords:** cytokine, T cell, idiopathic pulmonary arterial hypertension, connective tissue disease associated pulmonary arterial hypertension

## Abstract

Pulmonary arterial hypertension (PAH) is rare disease that is categorized as idiopathic (IPAH) when no underlying cause can be identified. Lungs of most patients with IPAH contain increased numbers of T cells and dendritic cells (DCs), suggesting involvement of the immune system in its pathophysiology. However, our knowledge on circulating immune cells in IPAH is rather limited. We used flow cytometry to characterize peripheral blood DCs and T cells in treatment-naive IPAH patients, compared with connective-tissue disease-PAH (CTD-PAH) patients and healthy controls (HCs). At diagnosis, T-helper (Th) cells of IPAH patients were less capable of producing TNFα, IFNγ, IL-4 and IL-17 compared to HCs. IPAH patients showed a decreased frequency of Th2 cells and significantly enhanced expression of the CTLA4 checkpoint molecule in naive CD4^+^ T cells and both naive and memory CD8^+^ T cells. Frequencies and surface marker expression of circulating DCs and monocytes were essentially comparable between IPAH patients and HCs. Principal component analysis (PCA) separated IPAH patients—but not CTD-PAH patients—from HCs, based on T-cell cytokine profiles. At 1-year follow-up, the frequencies of IL-17^+^ production by memory CD4^+^ T cells were increased in IPAH patients and accompanied by increased proportions of Th17 and Tc17 cells, as well as decreased CTLA4 expression. Treatment-naive IPAH patients displayed a unique T-cell phenotype that was different from CTD-PAH patients and was characterized by reduced cytokine-producing capacity. These findings point to involvement of adaptive immune responses in IPAH, which may have an implication for the development of therapeutic interventions.

## 1. Introduction

WHO group 1 pulmonary arterial hypertension (PAH) is defined as a condition with a mean pulmonary arteria pressure (mPAP) > 20 mmHg, normal left atrium pressure and pulmonary vascular resistance ≥ 3 Wood units [1]. PAH is a devastating condition with a high burden of disease. Patients with PAH are subdivided into subgroups based on different risk factors or underlying conditions, such as connective tissue disease (CTD). A special subgroup of PAH patients has no known risk factor or underlying disease and is therefore categorized as idiopathic PAH (IPAH). 

Currently, accumulating evidence is beginning to reveal an important role for the adaptive immune system in the pathogenesis and progression of PAH [2,3]. 

IPAH lung biopsies were shown to contain increased numbers of CD4^+^ T-helper (Th) cells, CD8^+^ cytotoxic T (Tc) cells and TCRγδ T cells, which were found in close proximity to blood vessels [4,5]. Likewise, increased peri-arterial Th cells were found in patients with schistosomiasis-associated PAH [6].

Regarding the various currently defined Th subsets (See Supplementary Table S1 for overview), particularly Th17 have been implicated in PAH pathogenesis. Th17 cells are known to play an important role in many inflammatory and autoimmune diseases [7,8] and were found in pulmonary tertiary lymphoid organs (TLOs) of IPAH patients [9]. Furthermore, activated Th cells of PAH patients expressed higher levels of IL-17 [10]. Th17 cells are an important source of the pro-inflammatory cytokines IL-17 and IL-22 and differentiate from naive Th cells in the presence of IL-1β, IL-6, and TGF-β [11]. The finding that both IL-1β and IL-6 are increased in the serum of IPAH patients compared to controls [12] would be in line with a role of Th17 cells in IPAH pathophysiology. Finally, we recently reported increased polarization of circulating follicular T helper (Tfh) cells towards a Th17-associated surface phenotype in IPAH patients [13]. A role for Tfh would also be supported by the finding of an increase in IL-21^+^ PD-1^+^ Tfh cells in TLOs and around pulmonary arteries of IPAH patients [9].

Next to the findings that implicate Th17 and Tfh cells, evidence is accumulating for compromised activity of regulatory T cells (Tregs) in the inflammatory milieu of PAH lungs. Aberrant Treg function is strongly correlated with a predisposition to PAH in patients and frequencies of Tregs were found to be increased in circulation [14,15,16]. An imbalance in Th17/Tregs cells influenced the prognosis in patients with CTD-PAH, pointing to clinical relevance of the Th17 and Tregs populations [17].

Opposite to lung tissue, in IPAH patients CD8^+^ T cells are decreased in circulation and present mostly with an effector memory phenotype [15,18].

Various recent findings support the involvement of B cells in IPAH. First, circulating plasma blasts are increased in IPAH patients [19]. Second, autoantibodies are present in approximately 40% of the IPAH patients [20]. These autoantibodies, recognizing endothelial cell surface antigens [21], are thought to be produced by plasma cells located within TLOs in IPAH lungs [9,19]. Third, we recently reported increased B cell receptor (BCR) signalling in circulating B cells in IPAH patients [13]. Moreover, we found that pulmonary injury in combination with enhanced B cell activation is sufficient to induce PH symptoms in mice [13].

Consistent with the involvement of adaptive immune responses in IPAH, also dendritic cells (DCs), which are critical for T cell activation, have been implicated in its pathology. In the lungs of IPAH patients, the numbers of conventional DCs (cDCs) and plasmacytoid DCs (pDCs) are increased, whereby pDCs were localised predominantly in the alveolar space in proximity to vessels [4]. In peripheral blood of IPAH patients, cDC numbers were decreased [22]. Together with the increase in pulmonary cDCs, this suggests enhanced migration into the lungs. In our mouse studies, specific activation of cDCs resulted in the development of PH symptoms, whereby cDCs were specifically localized in the lungs and right ventricle of the heart [23,24].

However, despite the evidence for their involvement in PAH pathogenesis, the characterization of circulating T cells and DCs in IPAH patients is limited, the effects of PAH-specific therapy on T cells have also not been investigated. Therefore, we aim to characterize the circulating T cell and DC compartment in patients with IPAH in detail. We investigate the cytokine-producing capacity, activation marker expression and subset distribution of both CD4^+^ and CD8^+^ T cells in treatment-naive IPAH patients and after one year of PAH-specific treatment. Next to healthy individuals, we analyse patients with CTD-PAH, who show clear involvement of the adaptive immune system, given the increase in Th17 cells [17] and the presence of autoantibodies [25]. Thus, we have the opportunity to identify possible overlapping pathophysiological features of the two PAH diseases.

## 2. Results

### 2.1. T Cells from Treatment-Naive IPAH Patients Show Reduced Cytokine-Producing Capacity

**Figure 1:**
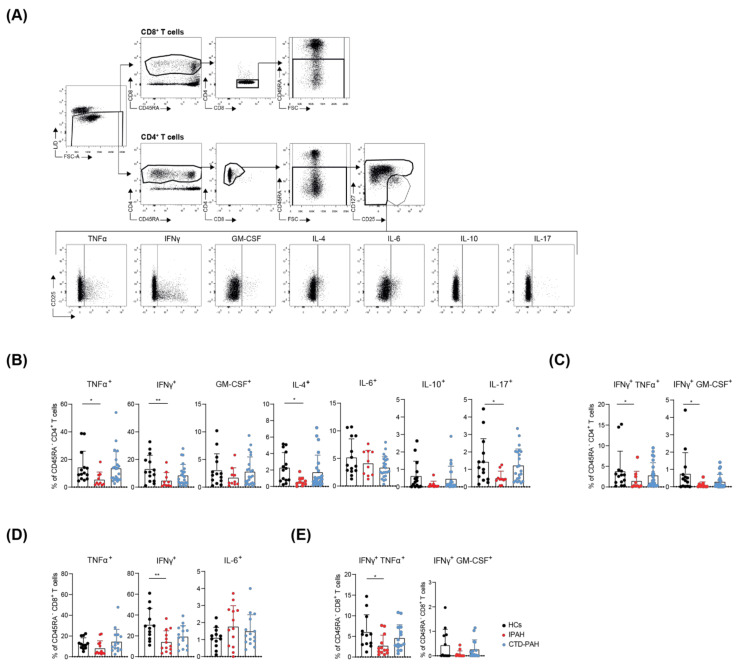
Circulating T cells of treatment-naive IPAH patients show reduced cytokine-producing capacity. (**A**) Flow cytometric gating strategy of cytokine production by circulating non-Treg CD45RA^−^ memory CD4^+^ T cells. (**B**,**C**) Quantification of the proportions of CD45RA^−^ CD4^+^ memory T cells producing the indicated cytokines in HCs, IPAH and CTD-PAH patients (**B**), with a subsequent quantification of the proportions of double-producers (**C**). (**D**,**E**) Quantification of the proportions of the cytokine-producing CD45RA^−^ CD8^+^ memory T cells in the indicated patients and HC groups (**D**), with a subsequent quantification of the proportions of double producers (**E**). (**A**–**E**) Samples with <500 events in parent gate were excluded from the analysis. Results are presented as mean + standard deviation; symbols represent value of individual patients or HCs. Mann–Whitney U test was used for statistical analysis, * *p* < 0.05, ** *p* < 0.01.

We investigated peripheral blood T cells in 15 treatment-naive IPAH and 24 CTD-PAH patients (Table 1), as well as 17 HCs. Cytokine production was analysed in CD45RA^−^ memory CD4^+^ and CD8^+^ T cell fractions, containing the T cells most prone to produce cytokines. Hereby, CD127^low^CD25^high^ CD4^+^ Tregs were excluded and analysed separately (see Figure 1A for the gating strategy and cytokine production of CD4 cells).

We observed that the memory CD4^+^ T cell fractions of IPAH patients had a significantly reduced cytokine-producing capacity for TNFα, IFNγ, IL-4 and IL-17, compared to HCs (Figure 1B). In addition, proportions of IFNγ/TNFα and IFNγ/GM-CSF double-producing CD45RA^−^ CD4^+^ T cells were reduced in IPAH patients, compared to HCs (Figure 1C). Likewise, frequencies of IFNγ single-producing and double-producing memory CD8^+^ T cells were lower in IPAH patients than in HCs (Figure 1D). In contrast, the cytokine-producing capacity of memory CD4^+^ and CD8^+^ T cells of CTD-PAH patients was not different from HCs (Figure 1B–E). Three of the CTD-PAH patients had immunomodulatory therapy, but their values for cytokine production were within the range of the other CTD-PAH patients analysed. The cytokines GM-CSF, IL-4, IL-10 and IL-17, which were produced by CD8^+^ T cells in lower amounts, did not significantly differ across the three groups analysed (Supplementary Figure S1).

Memory CD4^+^ and CD8^+^ T cells cannot only be divided into subsets on the basis of their cytokine production but also by specific chemokine receptor expression profiles. Using these profiles, we determined the T cell subset distribution in the two PAH patient groups and healthy controls and found that IPAH patients had reduced frequencies of Th2 cells (Supplementary Figure S2). Even though the Th1, Th17, and Tc1-associated cytokines IFNγ and TNFα were reduced, the proportions of Th1 and Th17 or Tc1 and Tc17 cells did not differ between IPAH and HCs (Supplementary Figure S2). By contrast, CTD-PAH patients displayed an increase in Th17 cells and a decrease in Th2 cells, whereas CXCR5^+^ (follicular) Tc, Tc17 and Tc1 fractions were reduced (Supplementary Figure S2). The proportion of Tregs did not differ between HCs and PAH patients (data not shown).

In conclusion, memory CD4^+^ and CD8^+^ T cells of IPAH patients were less capable of producing cytokines compared to HCs, whereas no differences were found in the cytokine-producing capacity of T cells of CTD-PAH patients.

### 2.2. T Cells from PAH Patients Show Increased CTLA4 Expression, Correlating with Cytokine-Producing Capacity

**Figure 2:**
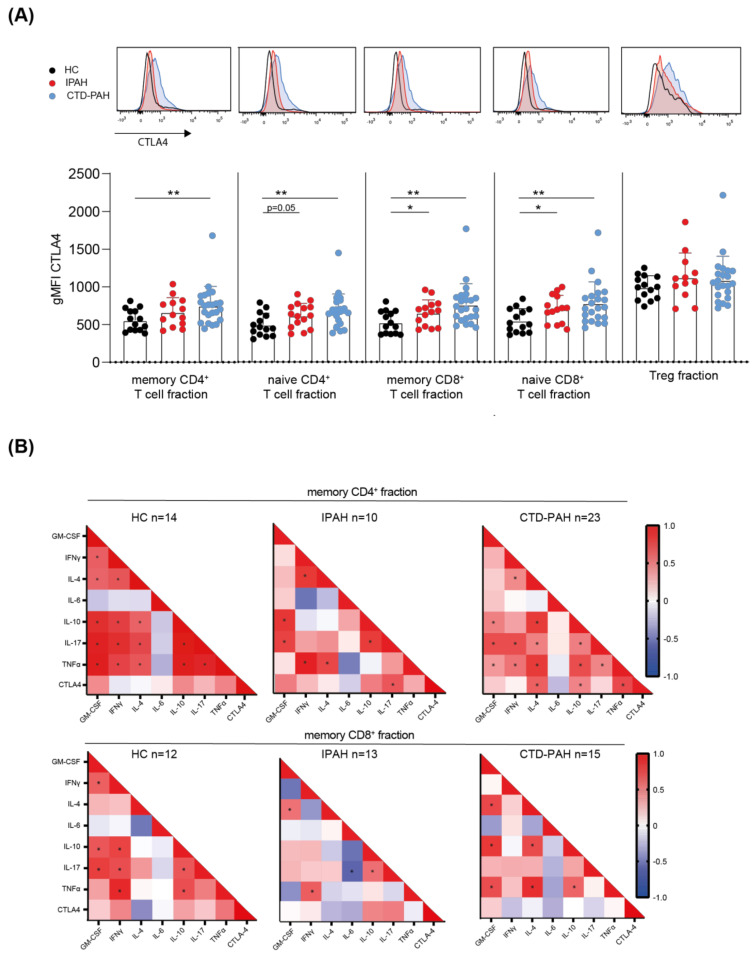
T cells of PAH patients show increased CTLA4 expression. (**A**) Quantification of intracellular CTLA4 expression, as determined by flow cytometry, in the indicated CD4^+^ and CD8^+^ T cells fractions of HCs, IPAH and CTD-PAH patients, shown as histogram overlays (**top**) and quantification (**bottom**). Samples with <500 events in parent gate were excluded (HC *n* = 14, IPAH *n* = 12–15 and CTD-PAH *n* = 22–23). (**B**) Associated correlation matrixes for cytokine-positive CD4^+^ and CD8^+^ T cells in HCs and patients with IPAH and CTD-PAH. Numbers of patients included are indicated above the correlation matrices. Correlation coefficient was calculated using nonparametric Spearman correlation. Results are presented as mean + standard deviation and symbols represent values of individual patients or HCs. Mann–Whitney U test was used for statistical analysis, * *p* < 0.05, ** *p* < 0.01. gMFI = geometric mean fluorescence intensity.

The reduced cytokine-producing capacity of T cells observed in IPAH patients might reflect an altered activation status and effector function of these cells. Therefore, we determined the expression of various surface markers on CD4^+^ and CD8^+^ T cells. Expression of the co-inhibitory receptor cytotoxic T lymphocyte antigen 4 (CTLA4) was significantly increased on naive CD4^+^ T cells and naive and memory CD8^+^ T cells in IPAH patients (Figure 2). For CTD-PAH patients, CTLA4 expression was increased in all CD4^+^ and CD8^+^ T cell fractions. Tregs, known to express high levels of CTLA4, showed similar expression across the three groups analysed. Expression of the activation marker T cell co-stimulator (ICOS) and the co-inhibitory receptor programmed cell death 1 (PD-1) expression did not differ between IPAH patients and HCs for both CD4^+^ and CD8^+^ T cells (Supplementary Figure S3).

Correlation matrices of the proportions of cytokine-expressing memory CD4^+^ T cells and CTLA4 showed a significant correlation with IL-17^+^ CD4^+^ T cells for IPAH patients and IL-4, IL-10 and TNFα for CTD-PAH patients (Figure 2B). In HCs, the expression levels of CTLA4 did not significantly correlate with the proportions of any of the cytokine-expressing memory CD4^+^ T cells in HCs. However, in HCs we found strong and significant correlations between the individual cytokines, except for IL-6, which demonstrated a weak, non-significant negative correlation with all other cytokines measured (Figure 2B). For IPAH patients, the analyses yielded a similar matrix of correlations between individual cytokines, although the observed correlations were more moderate, possibly due to a lower number of individuals included (*n* = 10, compared to *n* = 14 for HCs) (Figure 2B). The correlation matrix for CTD-PAH patients did not essentially differ from the matrix for HCs.

Memory CD8^+^ T cells of HCs also showed strong correlations, except for IL-4 and IL-6 (Figure 2B). In memory CD8^+^ T cells of IPAH patients, we observed fewer positive and more negative correlations, of which the correlation between IL-6 and IL-17 reached significance. Correlations between IL-17 and other cytokines lost significance in CTD-PAH patients. CTLA4 expression levels on CD8^+^ T cells were not significantly correlated with the proportions of positive cells for any of the cytokines in patients or HCs (Figure 2B).

Given that DCs are the main cell type that activate T cells and thereby induce expression of CTLA4 and ICOS, we next characterized DC subsets as well as monocytes, the precursors of inflammatory DCs, in peripheral blood. To this end, we investigated the myeloid compartment in a subgroup of PAH patients at diagnosis (12 IPAH and 17 CTD-PAH patients; see Supplementary Table S2 for patient details and Supplementary Figure S4A for gating strategy). The proportions of monocytes, cDCs, pDCs and AXL^+^ Siglec6^+^ (AS) DCs [26] did not differ significantly between IPAH patients and HCs (Supplementary Figure S4B–G). In addition, the activation status of DCs and monocytes, as indicated by the expression of CD86, CD80, HLA-DR and CD11c, was not altered, except for HLA-DR expression on classical monocytes which was moderately increased (Supplementary Figure S4H). In CTD-PAH patients, the size of the population of intermediate monocytes was slightly increased (Supplementary Figure S4C). The expression of the activation markers was increased to some extent on monocyte subsets in CTD-PAH patients, but DC subsets displayed a normal activation marker expression, as in IPAH patients (Supplementary Figure S4H).

In summary, except for naive CD4^+^ T cells in IPAH patients, the expression of CTLA4 was significantly increased in circulating naive and memory CD4^+^ and CD8^+^ T cells from both patient groups, compared with HCs. ICOS expression on CD4^+^ and CD8^+^ T cells was not affected, suggesting that their activation status remained unchanged. The cytokine correlation matrices revealed that in memory CD4^+^ T cells from PAH patients, CTLA4 expression correlated with cytokine production capacity, in particular with IL-17+ Th cells in IPAH. Furthermore, for both CD4^+^ and for CD8^+^ T cells, the correlations between cytokine expression were weaker in IPAH patients than in those in both HCs and CTD-PAH patients. Analysis of peripheral blood DCs and monocytes did not provide evidence for altered DC activation.

### 2.3. Principal Component Analysis of T Cell Cytokine Production Separates IPAH Patients from HCs

**Figure 3:**
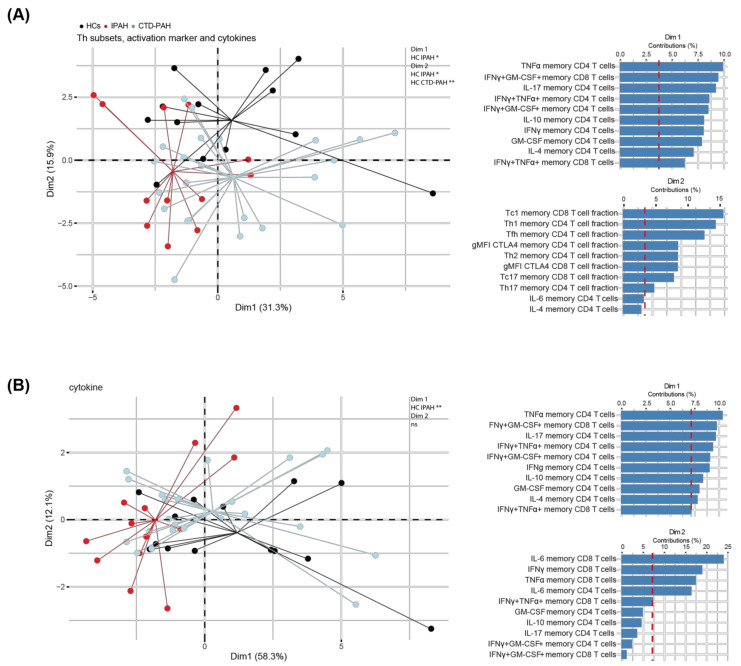
Multivariate analysis separates treatment-naive PAH patients from HCs, mainly by T cell cytokine production. (**A**) Principal component analysis (PCA) of HCs (*n* = 13), IPAH (*n* = 12) and CTD-PAH (*n* = 23) patients of whom all variables (peripheral T cell subsets, activation markers and cytokine production) could be determined by flow cytometry (**left**), with the contributions of the top 10 variables in percentages of Dim1 and Dim2 (**right**). (**B**) PCA of T cell cytokines only of HCs (*n* = 14), IPAH (*n* = 13) and CTD-PAH (*n* = 23) patients determined by flow cytometry and contributions of the top 10 variables in percentages of Dim1 and Dim2. Symbols represent values of individual patients or HCs, whereby lines connect these values to the mean Dim1 and Dim2 coordinates. Mann–Whitney U test was used for statistical analysis of coordinates on the dimension between PAH patients and HCs, * *p* < 0.05, ** *p* < 0.01.

Next, we investigated whether a multivariate analysis would be able to separate PAH patients and HCs, based the observed phenotypic differences in the T cell compartments. To this end, we performed a PCA using the obtained data of cytokine production, chemokine-receptor based Th subset distribution and activation marker expression (Figure 3A). IPAH patients were separated from HCs in the first dimension (Dim1; 31.3%), dominated by intracellular cytokine expression mainly in CD4^+^ T cells, and in Dim2 (15.9%), dominated by Th and Tc subset sizes and CTLA4 expression. CTD-PAH patients were separated in Dim2 only. Interestingly, a PCA solely based on frequencies of cytokine-producing cells could also separate IPAH patients from HCs but not CTD-PAH patients from HCs (Figure 3B). Hereby, the Th1- and Th17-associated cytokines contributed most to Dim1 (58%).

Taken together, these findings confirm that CD4^+^ and CD8^+^ T cells from treatment-naive IPAH patients have unique cytokine expression profiles that are significantly different from HCs.

### 2.4. T Cell Cytokine and CTLA4 Expression Profiles in IPAH Patients Significantly Change over Time

**Figure 4:**
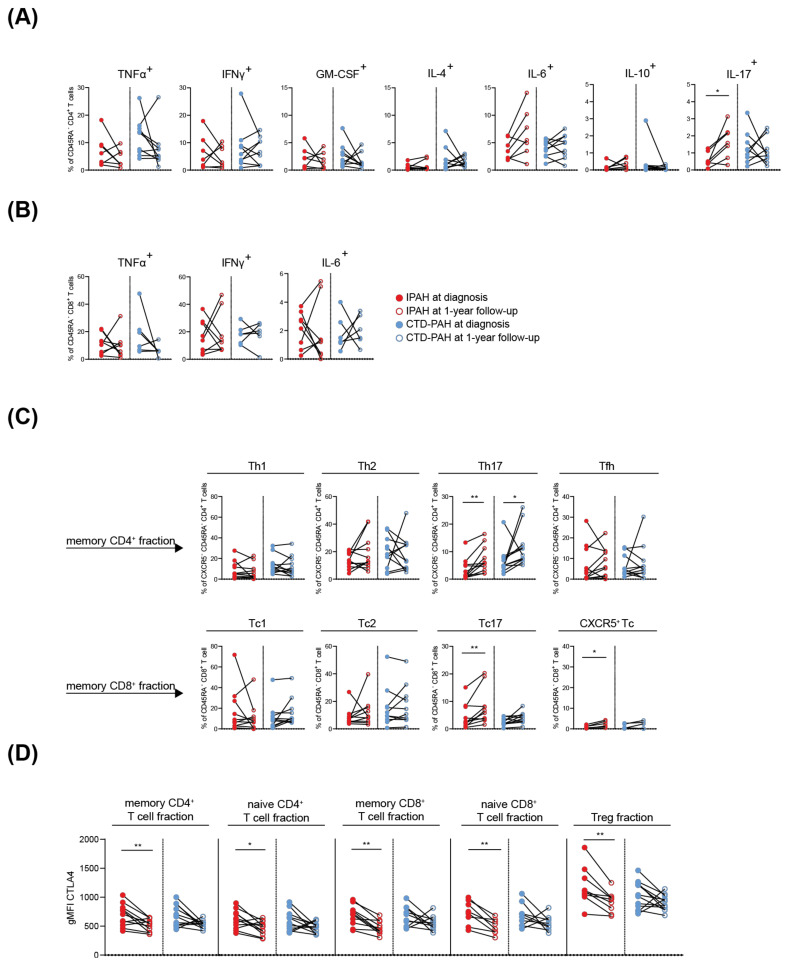
T cell profile changes over time in PAH patients. (**A**,**B**) Quantification of the proportions of CD45RA^−^ CD4^+^ memory T cells (**A**) and CD45RA^−^ CD8^+^ memory T cells (**B**) producing the indicated cytokines in paired samples from IPAH and CTD-PAH patients at diagnosis and at 1-year follow-up. (**C**) Proportions of peripheral blood CD4^+^ and CD8^+^ T cell subsets, based on chemokine receptor expression, in paired samples from PAH patients at diagnosis and 1-year follow-up. (**D**) Quantification of CTLA4 expression in the indicated T cell fractions in samples from IPAH and CTD-PAH patients at diagnosis and 1-year follow-up. Closed and open circles represent values of individual patients at diagnosis and 1-year follow-up, respectively. Paired samples are connected by a line. Wilcoxon matched-pairs signed-rank test was used for statistical analysis, * *p* < 0.05, ** *p* < 0.01. gMFI = geometric mean fluorescence intensity.

To determine the dynamics of the T cell cytokine and CTLA4 expression profiles in PAH patients, we analysed subgroups of 11 IPAH patients and 12 CTD-PAH patients of whom paired samples at diagnosis and 1-year follow-up were available for analysis. Whereas intracellular IL-17 in memory CD4^+^ T cells of IPAH patients increased over time, the other cytokines analysed remained stable (Figure 4A). The cytokine-producing capacity of memory CD8^+^ T cells of IPAH patients (Figure 4B and data not shown) and of memory CD4^+^ and CD8^+^ T cells of CTD-PAH patients (Figure 4A,B and data not shown) was unaltered over time.

The proportions of Th17 cells increased over time in both patient groups (Figure 4C). For IPAH patients, this rise of Th17 cells paralleled the observed increased frequency of IL-17^+^ CD4^+^ T cells over time. At 1-year follow-up, frequencies of CXCR5^+^ Tc or Tc17 cells were increased in IPAH patients but not in CTD-PAH patients.

The expression of CTLA4 decreased over time for all T cell populations in both patient groups, which only reached significance in IPAH patients (Figure 4D). Inferred from the values of the geometric mean fluorescence intensities, the CTLA4 expression at 1-year follow-up returned to the levels seen in HCs (compare Figure 4D and Figure 2A). ICOS expression on memory CD4^+^ T cells was significantly increased at 1-year follow-up in both patient groups (Supplementary Figure S5A). PD-1 expression remained stable in the memory CD4^+^ T cell fractions but decreased in memory CD8^+^ T cells, reaching significance for the CTD-PAH patient group only (Supplementary Figure S5B).

**Figure 5:**
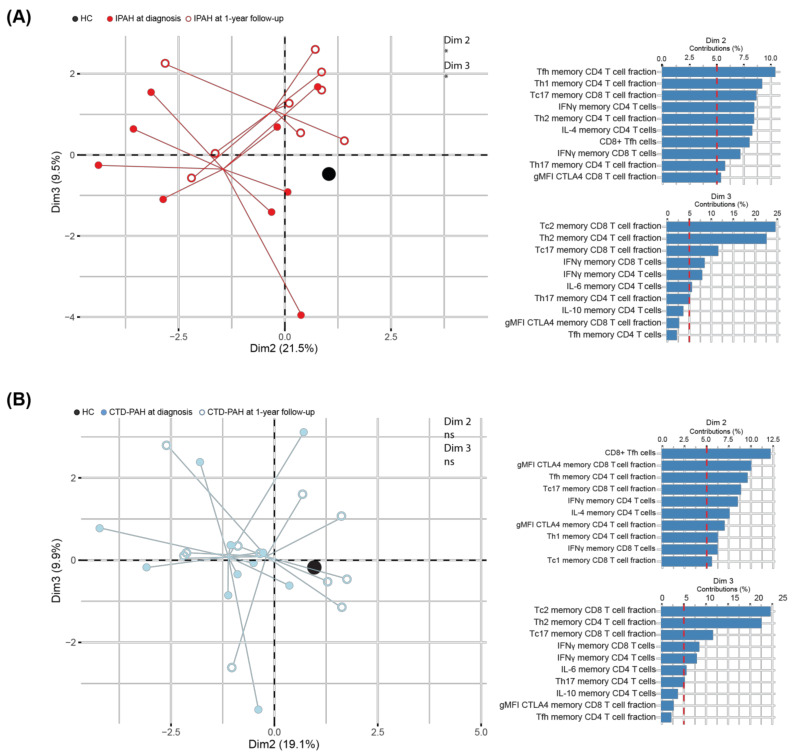
Multivariate analysis separates IPAH patients but not CTD-PAH patients at diagnosis and 1-year follow-up. (**A**,**B**) Principal component analysis (PCA) of IPAH patients (*n* = 9) (**A**) and CTD-PAH patients (*n* = 11) of whom all variables (peripheral T cell subsets, activation markers and cytokine production) could be determined by flow cytometry at 1-year follow-up (**left**), with the contributions of the top 10 variables in percentages of Dim2 and Dim3 (**right**). Symbols represent values of individual patients or HCs, whereby lines connect these values to the mean Dim2 and Dim3 coordinates. Mean coordinates of HCs are indicated in black. Wilcoxon matched-pairs signed-rank test was used for statistical analysis, * *p* < 0.05.

To obtain more comprehensive insight, we performed a PCA and found that IPAH patients at diagnosis and at 1-year follow-up were separated in Dim2 (21.5%) and Dim3 (9.5%), to which Th and Tc subsets, IFNγ^+^ T cells and CTLA4 contributed most (Figure 5A). Hereby, the IPAH patients at 1-year follow-up moved towards the profile seen in HCs in Dim2 but away from HCs in Dim3. Dim 1 (33.3%) did not separate IPAH patients at baseline and at 1-year follow-up. (Supplementary Figure S6A). Furthermore, our T cell parameters did not separate CTD-PAH patients at diagnosis from patients at 1-year follow-up (Dim2 and Dim3: Figure 5B; Dim1 and Dim3: Supplementary Figure S6B).

In summary, in IPAH patients the proportions of IL-17^+^ CD4^+^ T cells, Th17 and Tc17 increased at 1-year follow-up, compared to baseline. In both CD4^+^ T and CD8^+^ T cells, CTLA4 expression decreased, which was accompanied by increased ICOS expression for CD4^+^ T cells. Taken together, these dynamic changes suggest an increase in the activation status of T cells over time, with similar trends in IPAH and CTD-PAH patients. However, only in IPAH patients the T cell phenotype at diagnosis and at 1-year follow-up were separated in a PCA.

## 3. Discussion

The finding that the lungs of most patients with IPAH contain increased numbers of T cells and DCs suggested the involvement of the immune system in its pathophysiology [3,4,5,27] and prompted us to investigate these immune cells in peripheral blood. Using flow cytometry, we characterized circulating CD4^+^ and CD8^+^ T cells, DCs and monocytes in a well-defined cohort of treatment-naive IPAH patients, whereby results were compared to HCs and CTD-PAH patients.

Our analyses revealed that in IPAH patients, at diagnosis both the CD4^+^ and CD8^+^ T-cell compartment in peripheral blood contained reduced proportions of cytokine-producing cells and increased expression of the CTLA4 checkpoint molecule. We found a significant decrease for TNFα, IFNγ, IL-4 and IL-17 in CD4^+^ T cells and for IFNγ in CD8^+^ T cells, which separated IPAH patients from HCs in a PCA. After 1 year of PAH-specific therapy, proportions of IL-17^+^ CD4^+^ T cells, Th17 and Tc17 cells were increased, whereas CTLA4 expression on both CD4^+^ and CD8^+^ T cells was concomitantly decreased in IPAH patients. Our analyses showed that the phenotype of T cells in IPAH was different from CTD-PAH, in which cytokine expression was similar to HCs and did not change during 1-year follow-up. At baseline, CTD-PAH CD4^+^ and CD8^+^ T cells showed increased CTLA4 expression to levels that appeared even higher than those in T cells from IPAH patients.

The finding that the peripheral blood CD4^+^ and CD8^+^ T cell compartment of IPAH patients contained reduced numbers of cytokine-producing cells was striking. This concerned Th1-, Th2- and Th17-associated cytokines, and therefore we did not find evidence for a shift in Th subset ratios. The underlying mechanisms remain unknown but may involve T cell exhaustion, which is linked to increased CTLA4 expression [28]. However, this would not be supported by our finding that CTLA4 expression essentially showed positive correlations with proportions of cytokine-expressing cells. Moreover, in CTD-PAH patients, CTLA4 expression was also increased on CD4^+^ and CD8^+^ T cells, but their cytokine production was unchanged. Alternatively, the reduced cytokine production capacity could be due to other inhibitory signals.

Expression of PD-1, another co-inhibitory molecule associated with T cell exhaustion, and the activation marker ICOS on T cells remained unaltered in IPAH patients. Nevertheless, the specific increase in anti-inflammatory IL-10 in plasma or serum of IPAH patients (but not CTD-PAH patients) may well contribute to the observed differences in cytokine profiles [12,29]. It is also conceivable that cytokine-producing cells may have migrated into inflamed tissue, particularly because CD3^+^ T cells were found to be increased in lungs of IPAH patients [4]. Finally, it cannot be excluded that individuals with relatively low proportions of cytokine-producing cells in their circulation have an increased susceptibility to develop IPAH. In this context, the phenomenon may be related to findings in patients with chronic obstructive pulmonary disease (COPD). In the peripheral blood of these patients, significantly increased proportions of IFN-γ^+^ and TNF-α^+^ CD8^+^ T cells were only found in less severe disease cases but not in more advanced GOLD stage IV patients [30]. COPD patients with severely reduced diffusing capacity also had lower proportions of IL-17^+^ CD4^+^ T cells in their circulation.

In our analyses, the strong signals induced by PMA and ionomycin in vitro bypass the TCR-mediated signals supported by co-stimulatory signals dependent on the CD28/CTLA4 balance. Therefore, further experiments are required to determine the effect of increased CTLA4 expression on T cell activation and cytokine production in T cells from IPAH patients following TCR stimulation and co-stimulation in an interaction with antigen presenting cells. Hereby, it is of note that genetic variation in HLA-DPA1/DPB1 is associated with PAH [31], suggesting that interactions of CD4^+^ T cells with HLA class II-expressing cells, including monocytes, DCs or alveolar epithelial cells, may contribute to PAH development.

Because both IPAH and CTD-PAH are multifactorial diseases thought to develop by a multi-hit principle [32], it is attractive to speculate that the observed increased CTLA4 expression on T cells of both IPAH and CTD-PAH patients reflects one of the things that is common to the two diseases. In this context, it is of note that cytokine signatures, including TNFα, differentiate systemic sclerosis (SSc) patients at high versus low risk for PAH. However, it remains unclear whether immune changes contribute to the initiation of PAH symptoms in CTD or are a consequence of PAH. Another common factor might be Th17 cells, which have been implicated in various systemic auto-immune diseases, including SSc [33], as well as IPAH [10]. In addition, Tc17 cells or Tfh cells with a Th17-like chemokine receptor signature, which were increased in a heterogeneous group of IPAH patients [13], may contribute. Our finding that treatment-naive IPAH patients did not show increased frequencies of Th17 cells, whereas IL-17 production and Th17 cell frequencies increased at 1-year follow-up, would suggest that the Th17 phenotype develops during the disease. It remains unknown whether the increase in Th17 cells over time, as well as the increase in cytokine production in general, was a result of disease progression or PAH-specific medication. The former would be supported by the reported correlation between the frequencies of Th17 cells and disease severity in various diseases, including CTD-PAH [17] and psoriasis [34]. Overall, the groups of IPAH and CTD-PAH patients did not substantially differ in the specific PAH therapy they received. The increased cytokine production that we observed at 1-year follow-up in IPAH patients was not present in CTD-PAH patients, suggesting that this increase may be due to disease progression or disease-specific response to therapy, rather than a direct immunomodulatory effect of the PAH specific medication. However, effects of PAH-specific medication cannot be excluded, because these drugs have some immunomodulatory effects [35]. This may also explain the heterogeneity seen within the IPAH and CTD-PAH patient groups, whereby some patients show increased cytokine production capacity and others show decreased cytokine production capacity at follow-up. Particularly, because patients received either mono, duo or triple therapy with different classes of medication, perhaps exerting different effects on immune cells. A limitation of our study is the number of IPAH patients investigated. Because IPAH is a heterogeneous disease, it is very well possible that the immune profiles of subgroups of IPAH patients show a higher or a lower level of resemblance with CTD-PAH patients. Analysis of a larger group of treatment-naive IPAH patients could identify immune profile-based IPAH subgroups.

In IPAH patients, we did not observe major defects in peripheral blood myeloid cells, including DC subsets and monocytes. Yet, we previously showed spontaneous PH development in mice with a targeted deletion of the *Tnfaip3* gene in cDCs, resulting in aberrant DC activation [23,24]. Moreover, in human IPAH lung tissue we found co-localization of DCs and CD8^+^ T cells [23] and pDCs were more abundant in lung biopsies of IPAH patients [4]. Taken together, these studies provide evidence for a local involvement of DCs, which may exert a pathogenic role by initiation or maintenance of T cell activation, by maintaining lymphoid structures in the lung [36] and/or by initiating remodelling of pulmonary vessels [27]. The presence and activation status of DCs or monocytes in peripheral blood may not necessarily resemble their equivalents in the lung [37].

In conclusion, we found a significantly reduced cytokine-producing capacity of T cells of IPAH patients but not in CTD-PAH patients. The identified differences between IPAH patients and CTD-PAH patients in T cell subsets, their activation status and cytokine production capacity indicate different immune involvement across the two PAH subgroups. Although clinical trials treating PAH patients with immunomodulatory medication have been negative or inconclusive so far [38], our study illustrates the importance of a detailed understanding of the different immune phenotypes in the PAH subgroups for future interventional studies.

## 4. Materials and Methods

### 4.1. Subjects and Study Design

Thirty-nine PAH (15 IPAH and 24 CTD-PAH) patients were diagnosed according to the ERS/ECSC guidelines (Table 1) [39]. T cell activation, cytokine production and Th subset division was measured. In a subgroup of 29 patients (12 IPAH and 17 CTD-PAH), monocytes and DCs were analysed in addition (Supplementary Table S2). Similar to prior work from our group [29,40], exclusion criteria were: incomplete diagnostic work-up and therefore no confirmed PH diagnosis, not treatment-naive for PH therapy and age < 18 years or not capable of understanding or signing informed consent. Additionally, 17 HCs (41% female, mean age 55.3 ± 12.5) for T cell characterization and 12 HCs (42% female, mean age 50 ± 12.4) for myeloid characterization, were included. Exclusion criteria for HCs were: autoimmune disease, active infectious disease, use of immunomodulatory drugs and history of cardiopulmonary disease. The study protocol was approved by the Erasmus MC medical ethical committee (MEC-2011-392). Written informed consent was given by all patients and controls. The study was performed conforming to the principles outlined in the declaration of Helsinki.

### 4.2. Clinical Data Collection, Follow-Up and Definition of Endpoints

Hemodynamic and clinical data at diagnosis were collected during inpatient cardiopulmonary screening visits [29,40]. Data were collected and stored in PAHTool (version 4.3.5947.29411, Inovoltus), an online electronic case report form. Patients were treated according to the ERS/ESC guidelines [39] and prospectively followed up by half-yearly scheduled visits to the outpatient clinic.

### 4.3. Flow Cytometry

Peripheral blood mononuclear cells (PBMCs) were isolated—using ficoll density separation—from peripheral venous blood samples of PAH patients (taken directly after right heart catheterization) and HCs, using standard procedures. Cell counts were determined by manually counting using trypan blue and a Bürker Türk counting chamber. PBMCs were suspended in freezing medium containing 80% FCS and 20% DMSO and stored in cryogenic storage vials at −80 °C until further use. Flow cytometry procedures have been described previously [41] and antibodies used for intra- and extracellular staining are given in Supplementary Table S3. In brief, for the determination of T cell subsets, PBMC fractions were directly stained for the chemokine receptors and extracellular markers CCR4—FITC, CD45RA—PE TxR, CD4—PercP-Cy5.5, CXCR5—Pe-Cy7, ICOS—BV650, CXCR3—BV711, PD1—BV786, CCR6—APC, CD3—APC-Cy7, CD8—AF700 for 60 min at 4 °C in phosphate-buffered saline (PBS) supplemented with 5 mM EDTA and 1% BSA (MACS buffer). After fixation with paraformaldehyde (2% for 10 min at 4 °C) and permeabilisation step with saponin (0.5% for 30 min at room temperature), cells were intracellularly stained for FoxP3—PE and CTLA4—BV421. For DC/monocyte staining, PBMC fractions were stained for extracellular markers CD16—FITC, PD-L1—PE-CF594, CD56—Pe-Cy7, AXL—APC, CD3—AF700, CD19—AF700, CD20—AF700, CD86—Biotin, CD80—BV421, CD11c—BV605, CD123—BV650, HLA-DR—BV711, CD14—BV785 in MACS buffer for 30 min at 4 °C, after which cells were incubated with streptavidin—APC-Cy7 for 10 min at 4 °C. Subsequently, cells were stained after a fixation and permeabilisation step for IRF4—PE and IRF8—Percp-Cy5.5 for 60 min at 4 °C. For the measurement of cytokines, PBMCs were incubated for 4 h at 37 °C in RPMI Medium 1640 + GlutaMAX-I (Gibco) supplemented with 5% fetal bovine serum (Gibco, Billings, MT, USA), 10 ng/mL phorbol 12-mysristate 13-acetate (Sigma-Aldrich, St. Louis, MO, USA), 250 ng/mL ionomycin (Sigma-Adrich) and Golgistop (BD Bioscience, Franklin Lakes, NJ, USA), after which cells were stained for extracellular markers CD4—FITC, CD45RA—BV650, CD3—biotin, CD8—AF700, CD25—Pe-Cy7, CD127—BV421 and intracellular markers IL-10—PercP-Cy5.5, IL-4—PAC-Cy7, IL-6—PE, IFNγ—BV711, IL-17—BV786, TNFα—APC, GM-CSF—PE-TxR as previously described [42]. Non-specific labelling was prevented in all stainings by blocking Fc receptors using human TruStain FcX (Biolegend, San Diego, CA, USA) and dead cells were excluded with Fixable Viability Dye Live/Dead eF506 (eBioscience, Franklin Lakes, NJ, USA) and forward and side scatter values. Data were acquired using a FACSymphony A5 flow cytometer (Beckton Dickinson, Franklin Lakes, NJ, USA) and analysed using FlowJo v.10 (Tree Star Inc software, Ashland, OR, USA).

### 4.4. Principal Component Analysis and Statistical Evaluation

Principal component analysis (PCA) was performed using R and RStudio, and the packages FactoMineR and Factoextra, and this has been described previously [29,43]. Statistical evaluations of flow cytometry data and PCA dimension coordinates for differences between HCs and either IPAH patients or CTD-PAH patients were performed by Mann–Whitney U tests. Paired diagnosis and 1-year follow-up data were analysed by the Wilcoxon matched-pairs signed-rank test. Correlation coefficients were calculated using the nonparametric Spearman correlation. All statistical tests were two-sided; *p*-values < 0.05 were considered statistically significant. Statistical analyses were performed using GraphPad Prism v.8 (Graph Pad Software, San Diego, CA, USA).

## Figures and Tables

**Table 1:** Baseline demographic and patient characteristics.

	PAH—BASELINE			PAH—1Y FOLLOW-UP		
	IPAH (*n* = 15)	CTD-PAH (*n* = 24)	*p* Value	IPAH (*n* = 11)	CTD-PAH (*n* = 12)	*p* Value
**Baseline clinical characteristics**						
Gender, female (%)	13 (87%)	20 (83%)		10 (91%)	11 (92%)	
Age, y	55.6 ± 16.7	64.6 ± 11.2	0.15	60.8 ± 14.8	65.3 ± 12.2	0.59
BMI, kg/m^2^	28.4 ± 7.4	27.6 ± 5.4	0.86	29.1 ± 8.4	28.6 ± 6.1	0.96
NYHA class 3–4, *n* (%)	11 (73%)	14 (58%)		8 (73%)	7 (58%)	
6MWT, m	332 ± 126	299 ± 138	0.70	298 ± 121	334 ± 131	0.40
NT-pro BNP, pmol/L	242 ± 272	541 ± 1056	0.83	281 ± 308	301 ± 502	0.57
Underlying CTD						
SSc, *n* (%)		20/24 (83%)				
SLE, *n* (%)		4/24 (17%)				
**Baseline right heart catheterization**						
mPAP, mmHg	55.5 ± 15.2	44.0 ± 13.0	0.02	51.1 ± 12.7	41.5 ± 12.2	0.08
mRAP, mmHg	12.4 ± 6.3	9.5 ± 5.2	0.16	11.3 ± 6.5	8.3 ± 5.0	0.22
Capillary wedge pressure, mmHg	9.5 ± 4.8	13.5 ± 7.8	0.11	9.8 ± 5.3	13.6 ± 10	0.44
PVR, wood units	10.2 ± 3.1	6.2 ± 3.4	0.002	9.2 ± 3.0	5.7 ± 3.4	0.01
**PH-Medication**						
At baseline, *n* (%)	0/15 (0%)	0/24 (0%)				
At 1-year follow-up						
No PH-medication				0/11 (0%)	0/12 (0%)	
Mono therapy (ERA), *n* (%)				1/11 (9%) ^1^		
Mono therapy (PDE5), *n* (%)					1/12 (18%) ^2^	
Duo therapy (PDE5 + ERA), *n* (%)				6/11 (55%)	9/12 (73%)	
Triple therapy (PDE5 + ERA + PRC), *n* (%)				4/11 (36%)	2/12 (9%)	
**Immunomodulatory drugs**						
At baseline, *n* (%)	0/15 (0%)	3/24 (13%)				
At 1-year follow-up, *n* (%)				0/11 (0%)	3/12 (25%)	

^1^: This IPAH patient was on ERA monotherapy, due to severe side effects on PDE5 therapy. ^2^: This CTD-PAH patient was on PDE5 monotherapy due to severe side effects on ERA therapy. Data given as ‘mean, ± SD’, unless otherwise indicated. Abbreviations: BMI, body mass index; PAH, pulmonary arterial hypertension; IPAH, idiopathic pulmonary arterial hypertension; CTD, connective tissue disease; 6MWT, 6-min walk test; NT-pro BNP, The N-terminal prohormone of brain natriuretic peptide; SSc, systemic sclerosis; SLE, systemic lupus erythematosus; mPAP, mean pulmonary arterial pressure; mRAP, mean right atrium pressure; PVR, pulmonary vascular resistance, endothelin receptor antagonist; ERA, Phosphodiesterase 5 inhibitor; PDE5, prostacyclin; PRC.

## Data Availability

The data presented in this study are available in this article or upon reasonable request.

## Supplementary Materials

The following supporting information can be downloaded at: https://www.mdpi.com/article/10.3390/ijms23126508/s1. Refs. [8,11,44,45] are cited in Supplementary Materials.
