# Supplementary material for: Peripheral Blood T Cells of Patients with IPAH Have a Reduced Cytokine-Producing Capacity
Source: Int J Mol Sci. 2022 Jun 10;23(12):6508. doi: 10.3390/ijms23126508 (PMC9224379; doi:10.3390/ijms23126508)
Supplement: Supplementary file 1 [file ijms-23-06508-s001.zip › ijms-1735747-supplementary.pdf]

Table S1. Characteristics of CD4$^+$ Th subsets.

	CD4$^+$ T cell subsets				
	**Th1**	**Th2**	**Th17**	**Tfh**	**Treg**
Cytokines driving differentiation	IL-12, IFNγ	IL-4	IL-6, IL-23, IL-21, TGFβ	IL-21, IL-6, IL-27, IL-12	IL-2, TGFβ
Major function	Protection against intracellular pathogens	Protection against helminth infection	Protection against extracellular pathogens	Support to B cells in lymphocyte follicles	Maintaining immune tolerance
Pathological conditions	Autoimmunity	Allergy	Autoimmunity	Autoimmunity	Lymphoproliferative disease and autoimmunity
Key transcription factors	T-bet	GATA3	RORγt	BCL6	FoxP3
Key surface molecule	CXCR3	CCR4	CCR6	CXCR5	CTLA4
Effector cytokines	IFNγ	IL-4, IL-5, IL-13	IL-17, IL-22	IL-21, IL-10	IL-10, TGFβ

Abbreviations: Th, T-helper; IL, interleukin; IFNγ, interferon γ; TGFβ, transforming growth factor β; CTLA4, cytotoxic T lymphocyte antigen 4. References used: [1-4].

Table S2. Baseline demographic and patient characteristics.

**DC staining (baseline)**	**PAH - BASELINE**		
	**IPAH (n=12)**	**CTD-PAH (n=17)**	***p* Value**
**Baseline clinical characteristics**			
Gender, female (%)	11 (92%)	13 (76%)	
Age, y	57.2 ±18.3	65.8 ±11.2	0.37
BMI, kg/m2	26.5 ±4.7	26.1 ±5.4	0.71
NYHA class 3-4, n (%)	9 (75%)	11 (65%)	
6MWT, m	354 ±109	300 ±133	0.40
NT-pro BNP, pmol/L	236 ±301	650 ±1213	0.61
Underlying CTD			
SSc, n (%)		14/17 (82%)	
SLE, n (%)		3/17 (18%)	
**Baseline right heart catheterization**			
mPAP, mmHg	55.8 ±16.5	41.7 ±13.0	**0.008**
mRAP, mmHg	11.6 ±6.0	9.4 ±5.8	0.21
Capillary wedge pressure, mmHg	9.0 ±5.3	11.3 ±5.9	0.31
PVR, wood units	9.7 ±3.0	6.6 ±3.6	0.02
**Immunomodulatory drugs**			
At baseline, n (%)	0/12 (0%)	1/17 (6%)	

Data given as 'mean, ±SD', unless otherwise indicated. **Abbreviations:** BMI, body mass index; PAH, pulmonary arterial hypertension; IPAH, idiopathic pulmonary arterial hypertension; CTD, connective tissue disease; 6MWT, 6-minute walk test; NT-pro BNP, The N-terminal prohormone of brain natriuretic peptide; SSc, systemic sclerosis; SLE, systemic lupus erythematosus; mPAP, mean pulmonary arterial pressure; mRAP, mean right atrium pressure; PVR, pulmonary vascular resistance.

Table S3. Monoclonal antibodies used for flow cytometry.

Antibody	Conjugate	Clone	Company
CD4	FITC	Okt4	Biolegend
CD45RA	BV650	HI100	BD
CD3	Biotin	UCHT	eBioscience
CD8	AF700	SK1	Biolegend
CD25	Pe-Cy7	M-A251	BD
CD127	BV421	A019D5	Biolegend
Streptavadin	BV605	-	BD
IL-10	PCP	JES3-9D7	Biolegend
IL-4	APC-Cy7	MP4-25D2	Biolegend
IL-6	PE	MQ2-13A5	eBioscience
IFNγ	BV711	B27	BD
IL-17a	BV786	N49-653	BD
TNFα	APC	6401.111	BD
GM-CSF	PE TxR	BVD2-21C11	BD
CCR4	FITC	-	R&D
CD45RA	PE TxR	MEM-56	Life technology
CD4	PercPcy5.5	RPA-T4	Invitrogen
CXCR5	Pe-Cy7	MU5UBEE	eBioscience
ICOS	BV650	C3984A	Biolegend
CXCR3	BV711	1C6/CXCR3	BD
PD-1	BV786	EH12.1	BD
CCR6	APC	11A9	BD
CD3	APC-Cy7	UCHT1	Invitrogen
FoxP3	PE	236A/E7	Invitrogen
CTLA4	BV421	BNI3	BD
CD16	FITC	3G8	BD
PD-L1	PE-CF594	M1H1	BD
CD56	Pe-Cy7	B159	BD
AXL	APC	FAB154A	R&D system
CD3	AF700	UCHT1	eBioscience
CD19	AF700	HIB19	eBioscience
CD20	AF700	2H7	BD
CD86	Biotin	FUN-1	BD
CD80	BV421	L307.4	BD
CD11c	BV605	3.9	Biolegend
CD123	BV650	7G3	BD
HLA-DR	BV711	G46-6	BD
CD14	BV785	M5E2	BD
Streptavadin	APC-Cy7	-	eBioscience
IRF4	PE	3E4	eBioscience
IRF8	PercPcy5.5	V3GYWCH	eBioscience

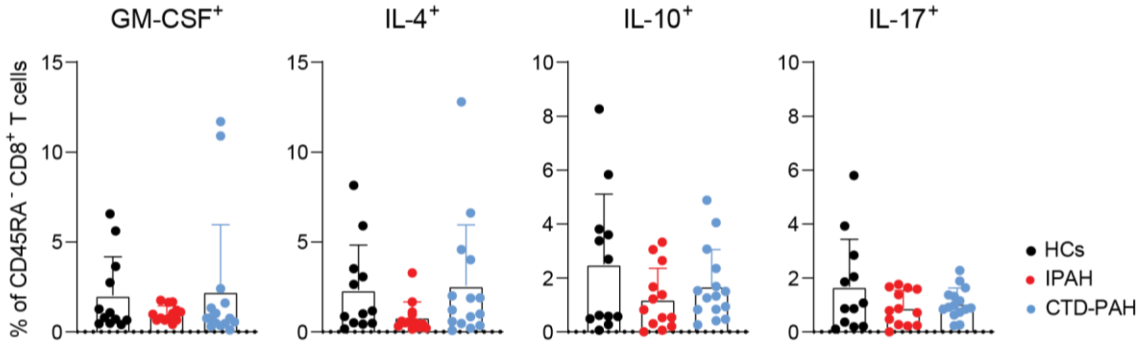

Figure S1. GM-CSF+, IL-4+, IL-10+ and IL-17+ memory CD8+ T cells in IPAH and CTD-PAH patients do not differ from HCs. Quantification of the indicated cytokines in CD45RA- CD8+ T cells. Results are presented as mean + standard deviation, Mann-Whitney U test was used for statistical analysis. Symbols represent values of individual patients or HCs.

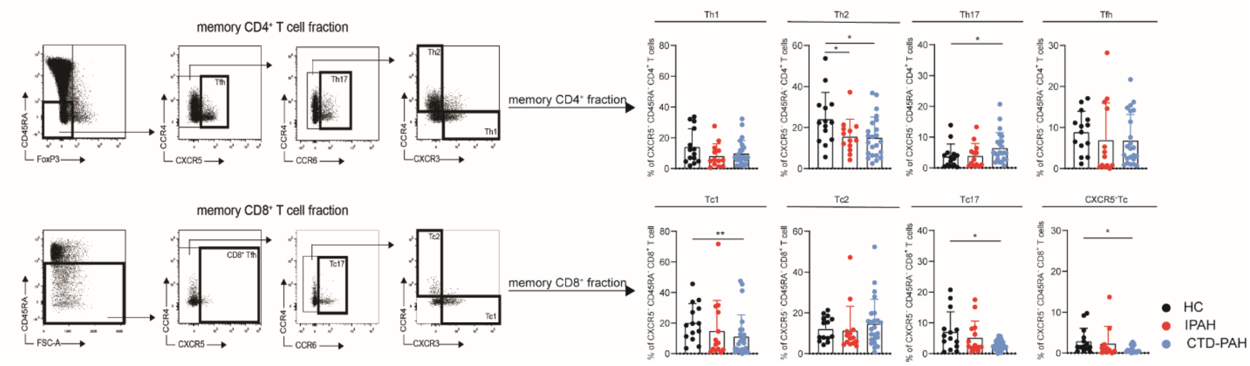

Figure S2. Frequency of Th2 cells is higher in PAH patients than in HCs. Gating strategy for peripheral blood Th subsets based on chemokine receptor expression (*left*) and percentages of circulating Th cells (*right*) of the indicated T cell subsets for HCs, IPAH and CTD-PAH patients at diagnosis, as determined by flow cytometry. Symbols represent values of individual patients or HCs. Results are presented as mean + standard deviation, Mann-Whitney U test was used for statistical analysis, * $p<0.05$, ** $p<0.01$.

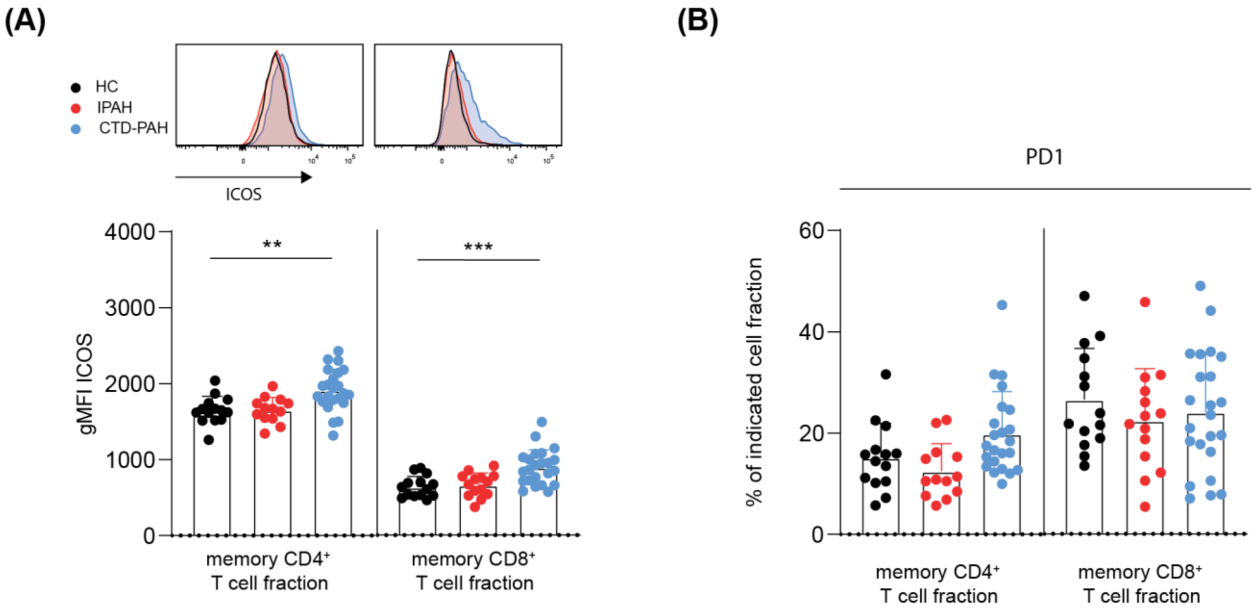

Figure S3. Increased ICOS expression on T cells of CTD-PAH patients. (A) Histogram overlays (*top*) and quantification (*bottom*) of ICOS expression, as determined by flow cytometry in HCs, IPAH and CTD-PAH patients. (B) Quantification of PD-1+ memory CD4+ and memory CD8+ T cells. Samples with <500 events in parent gate were excluded (HC n= 14, IPAH n=12-15 and CTD-PAH n=22-23). Results are presented as mean + standard deviation; symbols represent values of individual patients or HCs. Mann-Whitney U test was used for statistical analysis, ** $p<0.01$, *** $p<0.001$. gMFI = geometric mean fluorescence intensity.

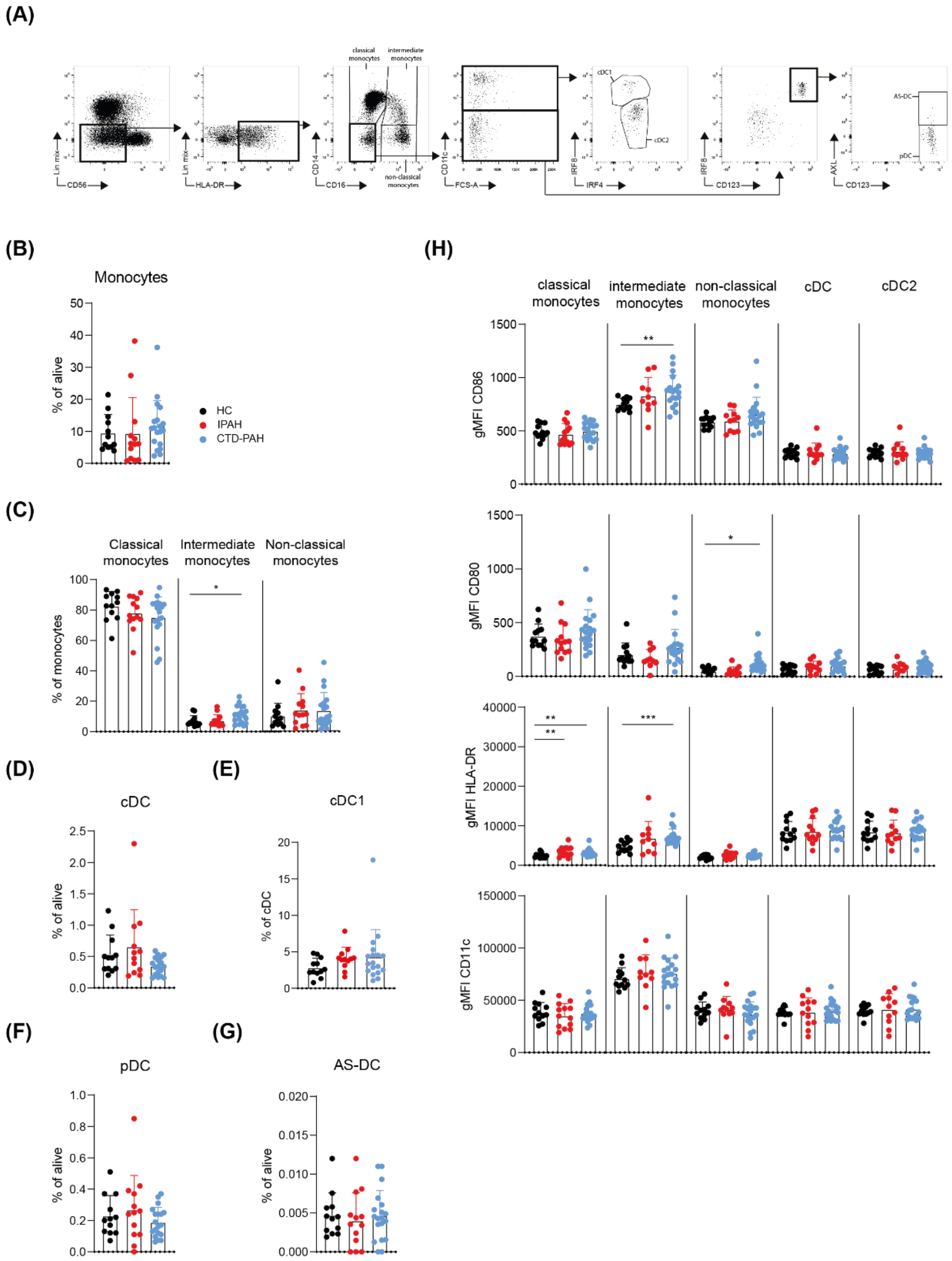

Figure S4. Limited differences in peripheral blood monocytes and dendritic cells between IPAH or CTD-PAH patients and HCs**.** (A) Flow cytometric gating strategy of monocyte and dendritic cell (DC) subsets. (B-G) Quantification of proportions of monocytes (B), monocyte subsets (C), conventional DCs (cDCs) (D), type 1 cDCs (cDC1) (E), plasmacytoid DCs (pDCs) (F) and AXL$^+$ Siglec$^+$ DCs (AS-DCs) (G). (H) Expression of the indicated activation markers on monocyte and DC subsets. Results are presented as mean + standard deviation; symbols represent values of individual patients or HCs. Mann-Whitney U test was used for statistical analysis, * p<0.05, ** p<0.01, *** p<0.001. gMFI = geometric mean fluorescence intensity.

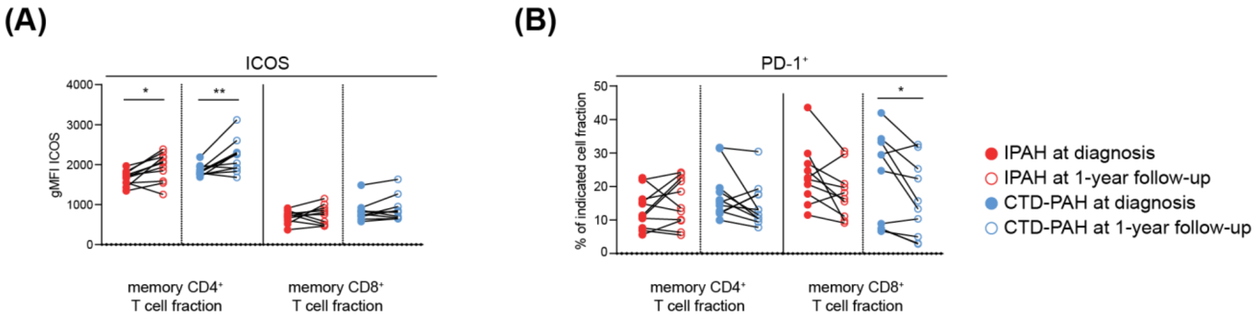

Figure S5. PD-1 and CTLA4 expression in PAH patients changes over time. (A-B) Quantification of ICOS expression (A) and PD-1 (B) in the indicated T cell fractions in samples from IPAH and CTD-PAH patients at diagnosis and 1-year follow-up, as determined by flow cytometry. Closed and open circles represent values of individual patients at diagnosis or 1-year follow-up, respectively. Paired samples are connected by lines. Wilcoxon matched-pairs signed rank test was used for statistical analysis, * $p<0.05$, ** $p<0.01$. gMFI = geometric mean fluorescence intensity.

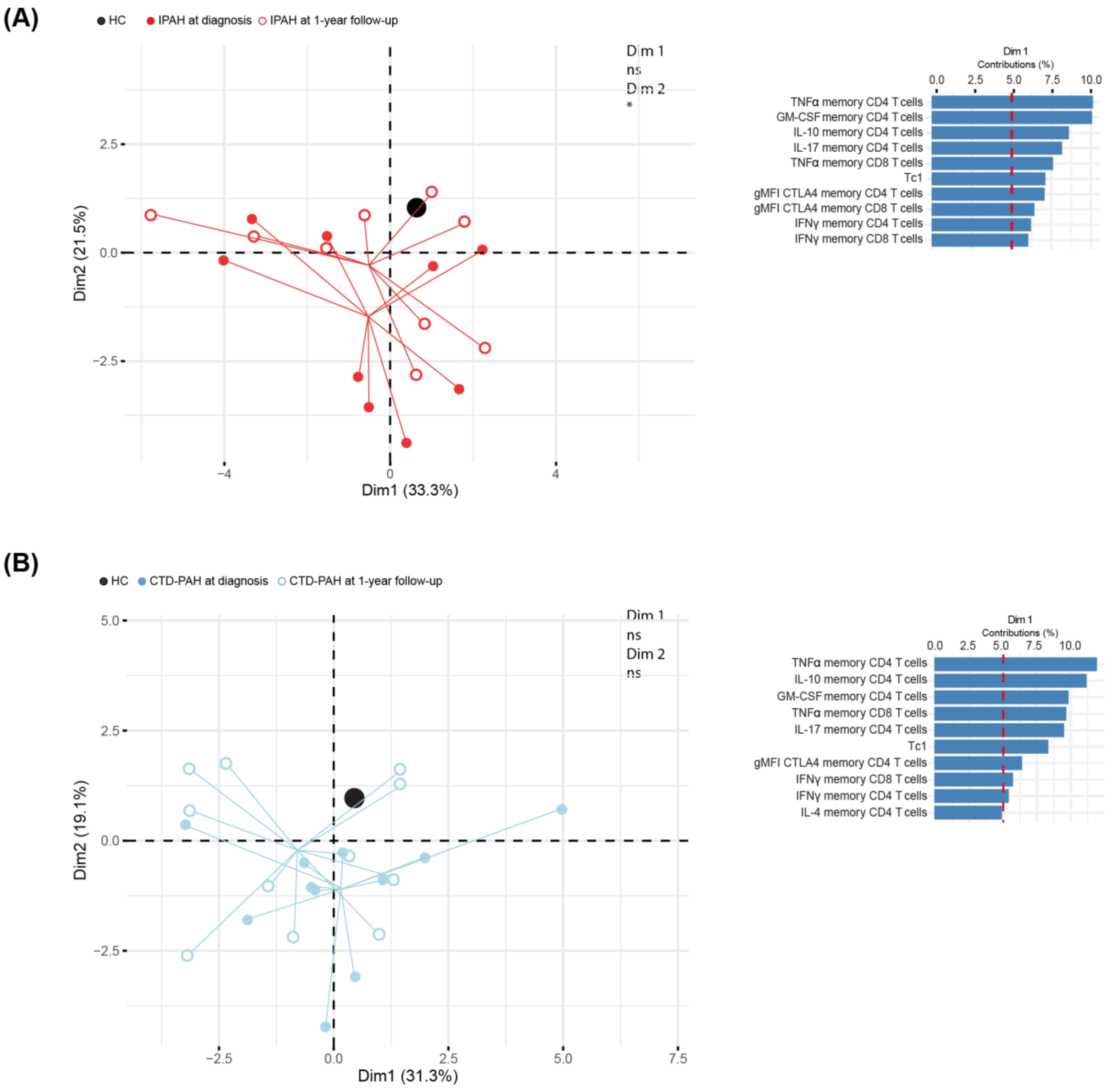

Figure S6. Multivariate analysis of IPAH patients and CTD-PAH patients at diagnosis and 1-year follow-up. (A-B) Principal component analysis (PCA) of IPAH patients (n=9) (A) and CTD-PAH patients (n=11) of whom all variables (peripheral T cell subsets, activation markers and cytokine production) could be determined by flow cytometry at 1-year follow-up (*left*), with the contributions of the top 10 variables in percentages of Dim1 and Dim2 (*right*). Symbols represent values of individual patients or HCs, whereby lines connect these values to the mean Dim1 and Dim2 coordinates. Mean coordinates of HCs are indicated in black. Wilcoxon matched-pairs signed rank test was used for statistical analysis. * $p<0.05$.

References

1. Stadhouders, R., E. Lubberts, and R.W. Hendriks, *A cellular and molecular view of T helper 17 cell plasticity in autoimmunity*. J Autoimmun, 2018. **87**: p. 1-15.
2. van Hamburg, J.P. and S.W. Tas, *Molecular mechanisms underpinning T helper 17 cell heterogeneity and functions in rheumatoid arthritis*. J Autoimmun, 2018. **87**: p. 69-81.

3. Tesmer, L.A., et al., *Th17 cells in human disease.* Immunol Rev, 2008. **223**: p. 87-113.
4. Rakebrandt, N., K. Littringer, and N. Joller, *Regulatory T cells: balancing protection versus pathology.* Swiss Med Wkly, 2016. **146**: p. w14343.
